# Strategies to reduce diagnostic errors: a systematic review

**DOI:** 10.1186/s12911-019-0901-1

**Published:** 2019-08-30

**Authors:** Julie Abimanyi-Ochom, Shalika Bohingamu Mudiyanselage, Max Catchpool, Marnie Firipis, Sithara Wanni Arachchige Dona, Jennifer J. Watts

**Affiliations:** 1 Deakin Health Economics, Centre for Population Health Research, Deakin University, Locked Bag 20000, Geelong, Victoria 3220 Australia; 2 Centre for Health Policy, Melbourne School of Population and Global Health, The University of Melbourne, 207 Bouverie St, Carlton, VIC 3053 Australia

**Keywords:** Diagnostic error, Audit, Communication strategies, Clinical setting

## Abstract

**Background:**

To evaluate the effectiveness of audit and communication strategies to reduce diagnostic errors made by clinicians.

**Methods:**

MEDLINE complete, CINHAL complete, EMBASE, PSNet and Google Advanced**.** Electronic and manual search of articles on audit systems and communication strategies or interventions, searched for papers published between January 1990 and April 2017. We included studies with interventions implemented by clinicians in a clinical environment with real patients.

**Results:**

A total of 2431 articles were screened of which 26 studies met inclusion criteria. Data extraction was conducted by two groups, each group comprising two independent reviewers. Articles were classified by communication (6) or audit strategies (20) to reduce diagnostic error in clinical settings. The most common interventions were delivered as technology-based systems *n* = 16 (62%) and within an acute care setting *n* = 15 (57%). Nine studies reported randomised controlled trials. Three RCT studies on communication interventions and 3 RCTs on audit strategies found the interventions to be effective in reducing diagnostic errors.

**Conclusion:**

Despite numerous studies on interventions targeting diagnostic errors, our analyses revealed limited evidence on interventions being practically used in clinical settings and a bias of studies originating from the US (*n* = 19, 73% of included studies). There is some evidence that trigger algorithms, including computer based and alert systems, may reduce delayed diagnosis and improve diagnostic accuracy. In trauma settings, strategies such as additional patient review (e.g. trauma teams) reduced missed diagnosis and in radiology departments review strategies such as team meetings and error documentation may reduce diagnostic error rates over time.

**Trial registration:**

The systematic review was registered in the PROSPERO database under registration number CRD42017067056.

**Electronic supplementary material:**

The online version of this article (10.1186/s12911-019-0901-1) contains supplementary material, which is available to authorized users.

## Background

Diagnostic error can be defined as “diagnosis that was unintentionally delayed (sufficient information was available earlier), wrong (wrong diagnosis made before the correct one), or missed (no diagnosis ever made), as judged from the eventual appreciation of more definitive information” [1] (page 1493). Diagnostic error as an area of patient safety has had insufficient research despite the costs in terms of negative health outcomes, loss of life, income and productivity, health system mistrust and dissatisfaction from both patients and health professionals [2, 3]. This has partly been attributed to the lack of an effective method to measure diagnostic errors, limited sources of reliable and valid data, and challenges of detecting diagnostic errors in clinical practice settings [4]. This is further complicated by diagnostic errors having many contributory factors at multiple levels of the patient care pathway, and diagnostic errors being context sensitive [5, 6]. Furthermore, diagnostic errors have differing definitions that make comparability across studies difficult [1, 7–11].

Earlier studies have mainly explored interventions to reduce diagnostic error including cognitive [12], system and process [13–18] errors. Regardless of the numerous studies on diagnostic errors [12–17], very few have investigated the effectiveness of strategies aimed at reducing diagnostic errors especially in a clinical setting [7, 19, 20], including audit and communication strategies. Clinical audit and communication strategies have been cited in the literature as a means to evaluate healthcare clinical performance, reduce diagnostic errors and improve quality of patient care [7, 21–24] 20). Graber et al., [18] and Singh et al., [25] emphasised that suggested approaches to diagnostic errors have rarely been operationalised in actual clinical practice hence there is a need to evaluate such interventions in the future.

To our knowledge audit and communication strategies to reduce diagnostic errors have not been studied separately. “Audit systems” were defined as systems that provide an individual or organisational performance measure against professional standards or targets to provide feedback to the individual or organisation [21–24]. This includes interventions such as processes, systems, models, programs and procedures aimed to ensure certain activities are carried out effectively and consistently to achieve the objectives [26]. Communication can be defined as the transmission of information and common understanding from one party to another [27]. The Committee on Diagnostic Error in Health Care supports processes for effective and timely communication between diagnostic testing, health professionals and treating health professionals and recommends that they should be implemented across all health care settings in the diagnostic process [7].

The aim of this systematic literature review is to summarize the current evidence on the effectiveness of audit and communication strategies undertaken by clinicians in reducing diagnostic errors within a clinical setting. This review will be helpful to clinicians that are involved in the diagnostic process; useful to managers in the clinical setting; and for policymakers involved in developing patient safety policies to improve the diagnostic process.

## Methods

### Search parameters and inclusion criteria

The systematic review follows PRISMA guidelines [28] and was registered in the PROSPERO database [29], registration number CRD42017067056. The search focused on audit and communication strategies implemented by clinicians in real patient or clinical environments to reduce diagnostic errors, with no restriction on the type of study design. Additional file 1 lists the details of the search strategies. We included articles written in English with sufficient information (at least an abstract).

The literature search included both published and unpublished work between January 1990 and April 2017. Database search included MEDLINE complete, CINHAL complete and EMBASE. Additional articles were manually searched using Agency for Healthcare Research and Quality Patient Safety Network (PSNet) [30] and Google Advanced search engine where unpublished studies were also located. In addition, systematic reviews retrieved from the database search were hand searched.

Two groups of two independent reviewers, (JAO and MF) and (SBM and MC), screened the titles and abstracts of articles from the databases to identify articles that met the inclusion criteria. Both eligible and inconclusive articles were included for full text screening. The same step was completed by the same groups for the articles from PSNet and a single reviewer (MF) screened the articles from the Google Advanced search engine. Articles that met inclusion criteria were added to the previously selected articles for full text review. Although both published and unpublished articles were included in the search, none of the unpublished articles met the inclusion criteria.

### Data extraction strategy

Information was extracted from each included study using a data extraction form that included: study population characteristics; descriptive information about study (year of publication, country, sample size, health states, study design, type of targeted clinicians); nature of the diagnostic error; nature of the intervention (technology based systems, additional patient reviews, staff education and training, structured process changes and specific patient examination instruments or forms); the effectiveness of interventions (as the difference between the intervention and the control) and nature of the clinical setting (emergency department, outpatients and primary care). All data extracted were crosschecked by the reviewers and any discrepancies discussed among the team until a consensus was reached.

### Quality assessment and risk of bias assessment

Study quality was assessed using the Cochrane Risk of Bias tool for RCTs (Randomized Control Trials) [31] and the Effective Public Health Practice Project quality assessment tool for non-RCT studies (observational descriptive, clinical trials, cohort/longitudinal and review) [32, 33]. Quality assessment data included selection bias, blinding of participants and researchers, blinding of outcome assessment, withdrawals and drop outs, selective reporting, data collection methods, study design, confounders, intervention integrity and data analysis. Studies were classified as high quality, medium quality and low quality. Publication bias and reporting bias on diagnostic errors as an outcome was minimised in this systematic review by inclusion of studies from multiple literature databases and searching unpublished “grey” literature.

## Results

### Study characteristics

**Fig. 1:**
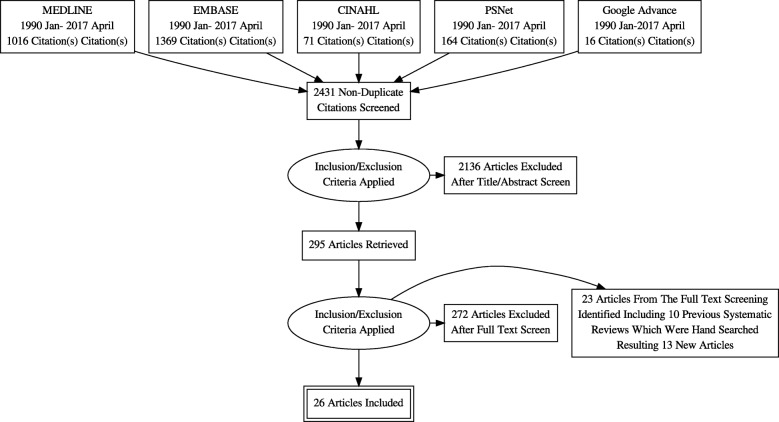
Literature Search PRISMA Flow Diagram- Systematic Review. Source: [34]

We identified 26 studies (Fig. 1) on strategies to reduce diagnostic error that met the criteria for full review. Nine studies (35%) were randomized controlled trials and the majority (17 of 26; 65%) had no randomisation, and were predominantly observational descriptive studies (9 of 26; 35%).

Twenty studies looked at audit systems [5, 35–53] and six studies considered communication strategies [54–59] employed by clinicians to reduce diagnostic errors. Nineteen studies were based in the US [5, 36–39, 41–44, 46, 47, 50, 51, 54–59]; 2 each in the UK [45, 52]; Sweden [40, 53]; Canada [48, 49]; and 1 from Lithuania [35]. Further details on study characteristics are given in Table 1.

**Table 1:** Diagnostic error prevention strategies

No	Author, year and country	Aim	Intervention delivery mode and strategy type	Design and sample size	Intervention	Control	Setting	Targeted Clinicians	Conclusion
	Communication Strategies								
1	Cannon et al., [54], 2000, US	To evaluate the effectiveness of computerised reminder system for mood disorder screening	Computerised trigger system via a communication strategy	RCT Total sample size =78 Intervention =41 Control =37	Computer program generated reminders to screen the patients for mood disorders. Program scored the mood disorder based on 4th edition of Diagnostic and Statistical Manual of Mental Disorders using answers given to questions patients’ progress notes were generated	Used a paper checklist inserted in the paper medical record	Psychiatric (outpatient)	Psychologists, registered nurses, social workers, therapists	Computer reminders shown to be superior to manual reminders in improving adherence to clinical practice guideline
2	Meyer et al., [56] 2016, US	To find effective communication strategies to inform primary care providers about the delayed follow-up	Computerised trigger system via a communication strategy	RCT Total sample size = 733 Control = 364 Intervention = 369	Communication through three escalating steps: 1st emails 2nd 3 phone calls 3rd inform clinical director	Usual communication process without any follow-up steps	Cancer	Physicians, physician assistants, nurse practitioners	Communication strategy to primary care providers on delayed follow-up of findings suspicious of cancer were useful, but not fail-safe. Need for additional back-up strategies - using case coordinators
3	Singh et al., [58] 2007, US	To examine the effectiveness of computerised notification system for diagnostic test results	Computerised trigger system via a communication strategy	Non-randomised descriptive study Total sample size = 1017	Weekly computerised tracking system to identify alerts of abnormal imaging reports	–	Laboratory	Health care providers, diagnostic-investigation providers	Computerised test result notification system alerted physicians of abnormal results through electronic medical record but imaging results continue to be lost to follow-up. Rate of results lost to follow-up lower than that reported in systems that do not use information technology
4	Medford-Davis et al., [55] 2015, US	To determine presence or absence of diagnostic error, detail of error and associated process breakdown underlying the errors	Computerised trigger system via a communication strategy	Non-randomised retrospective descriptive study Total sample size =100	An electronic “trigger” algorithm identified patients at high risk of diagnostic errors to facilitate selective record review	–	ED	ED clinicians	For patients in ED with abdominal pain, diagnostic process breakdown commonly involved history-taking, ordering insufficient tests in the patient provider encounter and problems with follow-up of abnormal test results
5	Murphy et al., [57] 2015, US	To determine if electronic patient record trigger system identifies patients at risk	Computerised trigger system via a communication strategy	RCT Total sample size = 72 Intervention = 36 Control = 36	Electronic triggers applied twice to electronic health record data repositories to identify records of patients with potential delays in diagnostic evaluation of findings suspicious for cancer	Usual follow-up without any electronic trigger activation	Cancer	Physicians, physician assistants, nurse practitioners	Electronic trigger-based intervention effective in reducing time to diagnostic evaluation of cancer. Intervention improved percentage of patients who received follow-up, can be used to improve timeliness of diagnosis of other serious conditions
6	Singh et al., [59] 2010, US	To examine whether notification alert system resulted in timely follow-up of abnormal laboratory results	Computerised trigger system via a communication strategy	Non-randomised prospective descriptive study Total sample size = 1163	Alert tracking system determined whether the alert was acknowledged the provider within two weeks of transmission; acknowledged alerts were considered read. Within 30 days of result transmission, record review and provider contact determined follow-up actions	–	Laboratory	Health care providers; diagnostic-investigation providers	Automated notification of abnormal lab results did not guarantee timely follow-up on non-life threatening abnormal lab results in the outpatient setting
	Audit Strategies								
7	Aaland et al., [5] 1996, US	To develop a policy to perform an ongoing series of patient examination during the entire trauma recovery process by a trauma team	Additional patient review via an audit system	Non-randomised descriptive study Total sample size = 1873	Patients evaluated in ED (Emergency Department) by trauma team and then discharged were followed within one week of the injury. New injuries identified were recorded and followed up	–	ED (Trauma)	General surgeons, ED physicians, medical students	Follow up can minimise diagnosis delays by: careful review of initial x-rays; repeating unclear studies; continued serial examination of each patient for entire clinical course; objectively and thoughtfully discussing missed injuries on a routine basis
8	Casalino et al., [36] 2009, US	To determine if a patient electronic medical records system reduced error rates	Additional patient review via an audit system	Non- randomised retrospective descriptive study Total sample size = 5434	A physician survey asked physicians about processes used by them to manage test results	–	Outpatient	Primary health care physicians	Failures to inform patients or to document informing patients of abnormal outpatient test results are common; use of simple processes for managing results was associated with lower failure rate
9	Perno et al., [44] 2005, US	Investigate delayed diagnosis of trauma while specific trauma team in place	Additional patient review via an audit system	Non-randomised prospective descriptive study Total sample size = 3265	Each paediatric trauma team member had a designated role in the evaluation and care of the trauma patient based on Advanced Trauma Life Support guidelines. After admission, each patient had a daily tertiary examination conducted by a trauma surgery physician starting within 24 h of initial evaluation	–	ED (Trauma)	ED paediatricians, ED surgeons, neuro surgeons, paediatric ICU (Intensive Care Unit) fellows, trauma nurses	Implementation of an effective paediatric trauma team associated with significant reduction in delay in trauma diagnosis
10	Selker et al., [47] 1998, US	To reduce number of cardiac care unit admissions without acute ischemia	Computer assistance via an audit system	Non-randomised controlled clinical trial Total sample size = 10,698 Intervention = 4738 Control = 5951	Acute Cardiac Ischemia Time-Insensitive Predictive Instrument (ACI-TIPI) automatically printed in patients ECG (Electrocardiogram)	Usual diagnosis using ECG without ACI-TIPI printed on	ED (Cardiology)	ED clinicians	ECGs with ACI-TIPI associated with reduced hospitalisation among ED patients without acute cardiac ischemia. ECGs with ACI-TIPI did not affect appropriate admission for unstable angina or acute infarction. Wide use ECGs with ACI-TIPI in the US is likely to lead to fewer unnecessary hospitalisations, especially to coronary care unit
11	Tsai et al., [51] 2003, US	To determine the effect of computerised ECG interpretation on non-cardiologists	Computer assistance via an audit system	RCT Total sample = 1620 Intervention = 810 Control = 810	Internal medicine residents interpreted two equally difficult ECG sets (Set A & B). First, they interpreted ECG set A without the computer interpretation support then interpret ECG set B with computer support.	Internal medicine residents interpreted ECG set B without the computer interpretation support first then interpret ECG set A with computer support	Laboratory (Cardiology)	Non cardiologists internal medicine residents	Computer decision support systems can generally improve the interpretive accuracy of internal medicine residents in reading ECGs
12	Bergman et al., [53] 2008, Sweden	To determine if novel diagnostic procedures improved diagnostic accuracy and proceeding time in psychiatry	Computer assistance via an audit system	RCT Total sample size = 63	Implemented a computer assisted diagnostic system to determine processing time and accuracy of diagnosis	Used paper and pencil method	Psychiatry	Clinical psychologists, general practitioners - specialists Specialist-clinical neurophysiology physicians	Results showed no major difference in diagnostic outcome between traditional paper and pencil methods and computer support for psychiatric diagnosis
13	Graber et al., [41] 2014, US	To reduce the likelihood of diagnostic error for patients presenting to ED	Checklist via an audit system	Non-randomised controlled trial Total sample size = 15	Used symptom specific checklist for high risk cases vulnerable for diagnostic error	Used a general checklist	ED	ED clinicians	Within the ED setting, checklists for diagnosis were helpful as they gave additional diagnostic possibilities and prevented diagnostic error
14	David et al., [37] 2011, US	To improve skin infection missed diagnosis using Visual-based computerised diagnostic decision support system	Computer assistance via an audit system	Non-randomised observational descriptive Total sample size =145	Used Visual-based computerised diagnostic decision support system (VCDDSS) to diagnose skin infection	–	ED (Dermatology)	ED clinicians	VCDDSS assisted primary care physicians to generate a more accurate diagnosis. Decision support tools should be included early in the diagnostic workflow to reduce misdiagnosis
15	Ramnarayan et al., [45] 2006, UK	To determine if a web based reminder system assisted junior doctors to improve diagnostic error	Computer assistance via an audit system	Non-randomised observational cohort study Total sample size = 8995 Diagnostic decision support systems access attempts =595	Junior physicians were given access to a web based diagnostic aid system to provide diagnostic assistance	–	Paediatrics	Junior physicians	A web-based diagnostic reminder system can successfully improve diagnostic decision making among junior doctors for acute paediatric assessments
16	Fridriksson et al., [40] 2001, Sweden	To educate local doctors to bring patients with subarachnoid haemorrhage to immediate neurological attention	Education program via an audit system	Non-randomised prospective descriptive study Total sample size =187	Seminars and individual referred case follow-ups were established monthly	–	Neurology	Local physicians to neurologists including nursing staff	Teaching programs focused on local physicians showed to have an impact on reducing diagnostic errors at low cost
17	Schriger et al., [46] 2001, US	To determine if computerised psychiatric interview could increase the mental disease detection in ED	Computer assistance via an audit system	RCT Total sample size = 190 Intervention = 92 Control = 98	Patients with complaints associated with occult psychiatric illness were asked to complete the Primary Care Evaluation of Mental Disorders (PRIME-MD) questionnaire in the ED waiting room and randomly assigned to intervention and control groups. Intervention group -PRIME-MD diagnosis results were given to physician	PRIME-MD diagnosis results were not given to the physician	ED (Psychiatry)	ED clinicians	Patients willingly completed the questionnaire (median time 7 min) which frequently diagnosed psychiatric conditions. However, physicians rarely diagnosed or treated these conditions regardless of being provided by PRIME-ED diagnoses
18	Wellwood et al., [52], 1992, UK	To increase accuracy in diagnosis of non-specific abdominal pain	Computer assistance via an audit system	Non-randomised cross over study Total sample size = 5193 Baseline: no diagnostic aid = 1610 Intervention 1 = 1598 Intervention 2 = 986 Intervention 3 = 999	Implementation of a computer aided system to increase accuracy in diagnosis of acute abdominal pain	–	ED (Gastrointestinal)	ED clinicians	Routine use of structured data collection sheets to collect details of acute abdominal pain need serious consideration; computerized systems increase accuracy
19	Espinosa et al., [39] 2000, US	To reduce clinically significant errors on radiographs interpreted in EDs	Additional patient review via an audit system	Non-randomised longitudinal study From, 1993 to 1994 = 28,161 1995 to 1996 = 20,236 1996 to 1999 = 67,111	ED physician performed immediate interpretation of all standard radiographs. A radiologist would provide an interpretation within 12 h as a quality control measure. Common errors in interpreting radiographs were discussed in a monthly meeting	–	ED	ED clinicians, radiologists	Error rates were reduced significantly using radiograph systems of interpretation to optimise clinician skills
20	Soininen et al., [50] 2012, US	To develop a versatile and objective computerised clinical decision support system for early detection of Alzheimer’s’ disease	Computer assistance via an audit system	Non-randomised observational descriptive study Total sample size = 400	A computer tool with composite disease indicators was implemented	–	Psychiatry - Alzheimer’s disease	Physicians	The tool provided objective information for early detection and prediction of Alzheimer’s disease using visualised patient data
21	Sibbald et al., [49] 2013, Canada	To determine if a checklist to interpret would improve diagnostic decision making	Checklist via an audit system	Non-randomised experimental control trial 15 clinicians interpreted 18 different ECGs under 4 conditions	Clinicians were asked to provide a summative interpretation of 18 different ECGs under four conditions: (i) undirected; (ii) verification without a checklist; (iii) verification with a checklist, and (iv) interpretation and verification with a checklist)	–	Cardiology	Cardiology fellows	Checklist use among ECG interpretation experts during the verification stage of diagnostic decisions did not increase cognitive load or cause expertise reversal, but reduced diagnostic error
22	Ely et al., [38] 2015, US	To test a diagnostic checklist for common symptoms	Checklist via an audit system	RCT Total physician sample size =14 Intervention =7 Control *n* = 7 Total patient sample size =10 Intervention =53 Control =47	A checklist was provided to physicians with differential diagnosis for common presenting symptoms in primary care	Usual diagnostic process	ED	Family physicians ED physicians	Checklists did not improve the diagnostic error rate in the study
23	Sibbald et al., [48] 2013, Canada	To evaluate checklists to improve cardiology diagnosis	Checklist via an audit system	RCT Total sample size = 191 Intervention =95 Control = 96	A simulator with six possible diagnosis was introduced. Residents examined the simulator as they examined a patient. Residents provided the diagnosis and estimate their certainty scale from 1 to 7. As the 2nd step intervention residents completed a checklist with re-examining the simulator	Follow the first step same as the intervention group but in the 2nd step, completed the checklist without re-examining the simulator	Cardiology	Internal medicine-residents	Verifying diagnostic decisions with checklists improved diagnostic accuracy. No evidence of increased cognitive load with use of checklists
24	Boguševičius et al., [35] 2002, Lithuania	To compare computer aided diagnostic accuracy with contrast radiography to diagnose acute small bowel obstruction	Computer assistance via an audit system	RCT Total sample size = 80 Intervention = 40 Control = 40	Developed computer program assisted clinicians to make differential diagnosis of the character of mechanical small bowel obstruction	Routine diagnosis without any computer aid	Radiology	Clinicians responsible for patient admission	Computer aided diagnosis was not superior to radiology contrast but needed significantly less time to perform diagnosis
25	Howard et al., [42] 2006, US	To implement a tertiary examination as standard care	Additional patient review via an audit system	Non-randomised observational prospective study Total sample size = 90	Introduction of a trauma tertiary exam form in addition to existing trauma history and physical examination forms	–	ED (Trauma)	Trauma nurse- specialists ED physicians	Suggested adoption of tertiary examinations as standard of care for patients admitted to level II trauma centres
26	Jiang et al., [43] 2000, US	To compare the effectiveness of independent double reading from computer support system and by radiologist	Computer assistance via an audit system	Non-randomised experimental study Total sample size = 104	Independent double reading and single-reading performance with a computer aid	–	Radiology	Radiologists	Computer aided diagnosis was an effective tool to improve clinical radiology practice

### Quality and risk of Bias assessment

**Fig. 2:**
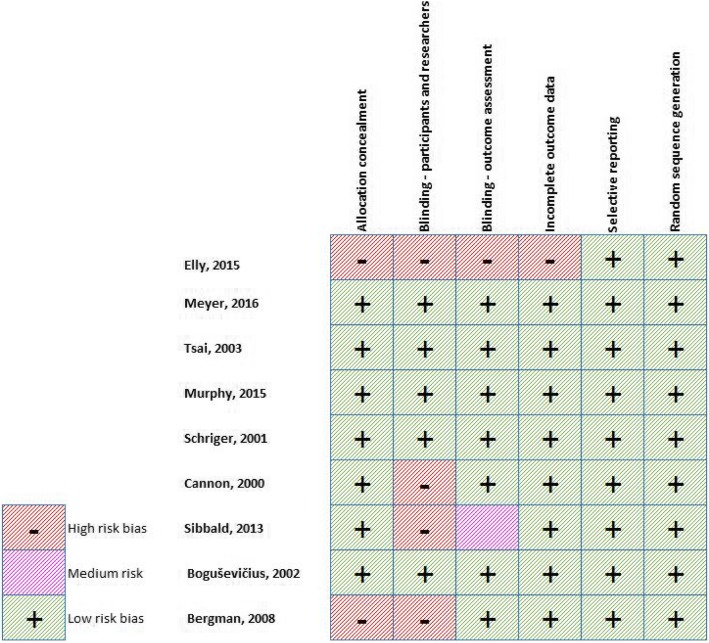
Risk of bias summary for RCTs

Results of the Risk of Bias assessment for RCTs is shown in Fig. 2 and Additional file 2.1. Two studies had selection bias due to allocation non-concealment, four studies demonstrated high Risk of Bias due to non-blinding and two studies rated as medium to high Risk of Bias due to non-blinding of assessment outcome. In summary there were 9/54 (16%) criteria assessed as medium to high Risk of Bias across all 9 RCTS and five of the nine studies were assessed as low Risk of Bias on all criteria. This suggests the quality of the RCT studies is relatively high.

Results of quality assessment for the non-RCT studies are shown in the Additional file 2.2. The quality of these studies was medium quality with most rated as weak for non-randomized study design.

### Strategies to reduce diagnostic error

Included publications were summarized under communication strategies and audit processes. These were further analysed by the types of communication or audit processes, disease group, healthcare setting and/or target clinician group.

### Communication strategies

Six studies examined the interventions related to communication strategies to address diagnostic errors [54–59]. There was one study in an emergency setting (abdominal pain) [55], two studies in primary care settings (cancer) [56, 57] and three studies in an outpatient setting (psychiatry and laboratory) [54, 58, 59]. The communication interventions were technology based systems, mostly computerised trigger systems.

Our review located three recent studies that examined trigger algorithms to identify patients with potential delayed diagnosis or follow-up in order to reduce diagnostic errors [55–57]. Murphy and his team [57] tested an algorithm in a randomised controlled trial to identify patients at risk of delays in diagnostic evaluation for a range of cancers. The intervention effectively reduced time to diagnostic evaluation and increased the number of patients that received follow-up care. Another RCT [56] identified follow-up delays via an electronic health record based algorithm and record reviews that communicated information through three alert steps: email, telephone call to clinicians, and informing clinical directors. This intervention led to more timely follow-up and diagnosis. However, effectiveness was reduced by non-responsiveness of clinicians in relation to triggers which meant that back-up strategies were required. Medford-Davis and colleagues’ algorithm [55] identified patients at high risk of delayed diagnosis or misdiagnosis who presented at the emergency department with abdominal pain and returned within 10 days requiring hospitalisation. This study concluded that breakdown in diagnostic processes led to diagnostic errors, finding that triggers provided opportunities for process improvement within emergency departments.

There were three studies that used computerised notification systems either as reminders or alerts for abnormal lab test results for timely follow-up to reduce diagnostic errors. Cannon and Allen [54] in an RCT compared effectiveness of a computer reminder system with a manual reminder system in terms of adhering to the implementation of clinical practice guidelines and found the computer system to be more effective. However, Singh and colleagues [59] in a prospective study revealed automated notifications of abnormal laboratory results through electronic medical records were unable to guarantee timely follow-up. Similarly, another study by Singh and colleagues [58] used a computerised test result notification system to reduce errors in communication of abnormal imaging results however the intervention was unable to prevent results from being lost to follow-up. Neither of these studies were RCTs.

### Audit processes

Twenty studies examined the interventions related to audit to address diagnostic errors [5, 35–53]. There were 10 studies in emergency settings (including two trauma and two cardiology) [5, 37–39, 41, 42, 44, 46, 47, 52], one in an outpatient setting [36], three in laboratory settings [35, 43, 51], and two in hospital setting [45, 48]. Four studies did not explicitly mention the setting [40, 49, 50, 53].

### Additional patient reviews

There were five studies on additional patient reviews [5, 36, 39, 42, 44]. Two studies examined use of trauma teams to diagnose complex injuries in trauma patients [5, 44] and showed use of a trauma response team reduced delayed diagnosis. One US study examined the impact of tertiary examination, a complete re-evaluation, on missed diagnosis of injury at a Level II Trauma Centre [42] and revealed 14% missed injuries, hence recommended adoption of this intervention as standard care at Level II Trauma Centres to improve accuracy of injury diagnosis. Another study [39] used a three pronged strategy for improving the diagnostic interpretation of radiographs that used a combination of review at monthly meetings, documenting errors and ongoing training of new staff and found a significant reduction in error rates. A study by Casalino and colleagues [36] audited 23 primary care practices using retrospective medical record review to determine if patients had been informed when test results were abnormal. Practices with partial electronic medical records were found to be less likely to inform patients of abnormal results compared to fully paper-based, or fully electronic systems.

### Computerised decision support systems

Eleven studies were based on computerised decision support systems [35–37, 43, 45–47, 50–53]. Studies by Tsai and colleagues [51], and David et al. [37] showed improvement in diagnosis accuracy using computer-based interpretation. Support systems enhanced junior doctor’s ability to diagnose acute paediatric conditions [45]; increased accuracy in diagnosis of acute abdominal pain [52]; and provided more accurate prediction of Alzheimer’s disease [50]. Ramnarayan and colleagues [45] stated that eliminating barriers to computer access is crucial for computerised assistance in clinical settings for the improvement in diagnosis. Boguševičius and colleagues [35] compared diagnosis of acute small bowel obstruction using computer aided diagnosis with radiology contrast, whilst they found no difference in accuracy, the computer aided time to diagnosis was only 1 h compared to 16 h for contrast radiology. Jiang et al., [43] compared a single radiologist reading, independent double reading by two radiologists and single reading with computer aid. They found computer aided diagnosis superior to all other methods in improving diagnostic accuracy of radiology reports.

One study found a computer diagnostic system to improve diagnosis of occult psychiatric illness but found no guaranteed response from the physician to diagnose or treat the condition [46]; and another found no difference in missed diagnosis of mental health conditions comparing computer aided diagnosis with traditional pen and paper [53]. Both studies favouring the traditional method for difficult mental health cases. Selker and colleagues [47] showed that computerised prediction did not impact on admission of people with acute cardiac ischemia but reduced unnecessary admission of people without the condition.

### Checklists

Checklists were used in four studies [38, 41, 48, 49]. Graber and his colleagues [41], used checklists in emergency settings and concluded that checklists could prevent diagnostic errors because they included additional diagnostic possibilities, however the study indicated the need to consistently use the checklists in collaboration with patients to achieve maximum value in usage of checklists.

Two Canadian studies showed improvement in accuracy of diagnosis in cardiology using a checklist approach: one used a checklist in verification of diagnosis by experts [49]; and another reviewed a cardiac exam using a checklist [48]. The third study used a checklist of symptoms but diagnosis accuracy was not different from usual care [38].

### Education programs

One study that was based on education programs [40] in primary care settings showed evidence of improved diagnostic accuracy through training and the use of a standard questionnaire. This study showed a 77% reduction in diagnostic errors due to an ongoing education program between physicians and neurosurgeons.

### Effectiveness of audit and communication strategies

The 9 RCTs were explored to determine the effectiveness of the interventions in reducing diagnostic errors. Three studies(54, 56, 57)were on communication and 9 on audit strategies [35, 38, 46, 48, 51, 53].

Cannon and colleagues [54] found the rate of screening increased by 25.5% for a reminder system compared to a checklist in a psychiatric outpatient setting. Another study [57] in a primary care setting (cancer) showed that patient identification triggers in combination with communication to primary care providers reduced the time to diagnostic evaluation by 96, 48 and 28 days for colorectal cancers, prostate cancers and lung cancers respectively. In addition, 21.2% more patients received diagnostic evaluation by the primary care providers’ final review. Meyer and colleagues [56] examined 3 escalating communication strategies-first emails, followed by telephones and lastly contact by clinic directors in reducing delayed follow-up using the same study by Murphy and colleagues [57]. Delayed follow-up was 88.9% using email, 54.5% for contact by clinic directors, and 31.4% using telephone.

Tsai et al., [51] reported that computer assistance in a laboratory setting increased the accuracy of interpretation of electrocardiograms by 6.6%, therefore reducing wrong diagnosis. Checklists used for audit process were found to increase correct diagnosis by 5% in a hospital setting [48]. Another study [38] revealed a diagnostic checklist made no difference in diagnostic errors among primary care physicians although there was a reduction of 25.9% among emergency physicians sub-group. However, three studies [35, 46, 53] identified computerised decision support systems to have no effect on the frequencies or the accuracy of diagnosis. Further details of the effectiveness of the interventions in non-RCTs is provided in Additional file 3.

## Discussion

This is the first systematic review on clinician focused audit or communication strategies employed to reduce diagnostic errors in real clinical practice settings. Twenty-six studies on strategies to reduce diagnostic errors were reviewed. The majority of studies were US based (19 studies), and high quality trials in terms of RCTs were low (9 studies, 35%). There were no studies that considered additional benefits to providers or clinical practices such as cost effectiveness or return on investment.

Our results confirmed earlier research [18, 25] by highlighting that there are very few systems that improve diagnostic error rates in real practice settings despite there being substantial information on the significant impact of diagnostic errors. To help address this gap, there is an urgent need for future research to evaluate such interventions to establish their effectiveness and cost effectiveness in actual practice.

The bias towards studies from the US may limit the generalisability of interventions to address diagnostic errors. Of the studies from the US, 8 (42%) were based in the ED, which may further impact on generalisability of findings. The organisation and funding of health care in the US varies considerably to other jurisdictions, with prevalence of private insurers impacting care as a major stakeholder in the system. Investment in high quality research beyond the US is warranted so that comparability with other countries and health systems is feasible.

The interventions in our study were mostly technology-based systems (*n* = 16, 62%) mainly computer decision support systems and alert systems. Technological advancements have meant that decision support systems are more likely to be available to clinicians. Nearly all computer decision support systems demonstrated improvement in the diagnostic process. However, it is vital to consider the barriers to technical access [45], including technical capacity of organisations and clinicians; and how effectively decision support systems can be integrated within the existing capacity of organisations [60] to realise the benefits in reducing diagnostic errors.

Technology based interventions reduced clinician bias by prompting clinicians to consider a variety of conditions that might be relevant to a patient’s clinical presentation. Our review revealed twofold improvement in the rate of accurate diagnosis through the use of checklists for cardiac examination [48], and improvement in the overall diagnostic process by shortening the time to diagnosis, for example 16-fold quicker time to diagnosis of acute small bowel obstruction compared to radiology contrast [35].

Patient safety research has highlighted the lack of appropriate measurement information for diagnostic errors hence the difficulty to ascertain the frequency of occurrence relative to other medical errors [7]. Studies identified in our review had outcome measures that varied significantly, including rates of screening [54], time to diagnostic evaluation [35, 44] and lost to follow-up rates [56, 58, 59]. Although there is ‘no one size fits all approach’ to measuring diagnostic errors improving the methods of identification of such errors will also improve measurement information.

Feedback to clinicians on their errors has the potential to improve the overall diagnostic process and therefore patient safety [61, 62]. Our review showed evidence of radiologists benefiting from error review [43], however this will depend on an organisational culture that is open to sharing information from their data sources.

Changing the culture of organisations in relation to diagnostic errors where the focus on feedback and diagnostic performance is correction of the system (using non-litigation approaches) and learn from diagnostic errors rather than focus on the individual who made the error has been suggested as a means to improve the learning process of clinicians [61, 63, 64]. Results from our review did not detect any culture change interventions for diagnostic errors.

Education and training interventions have been highlighted to improve the diagnostic process, our review identified only one study that explored the impact of education on diagnostic error rates [40]. Broadening the composition of the healthcare team improved accuracy in the diagnostic process through greater consultation and discussion between healthcare professionals, for example a paediatric specialist trauma team was shown to significantly reduce delay in trauma diagnosis [44].

Realising the full benefit from an intervention requires clinicians to be responsive to any additional information received from the intervention. There was evidence of improvement in the diagnostic process for some of the tested interventions but the benefit was only realised when clinicians accepted and acted upon the recommendations given [46, 56, 58, 59]. Clinician’s unresponsiveness to provided information limits realisation of benefits to the patient, hence the need for back-up strategies to improve physician responsiveness and therefore intervention effectiveness.

### Strengths and limitations of review

The strengths of the review include use of two independent reviewers which controlled for random errors and bias in deciding included studies [65, 66]; searching the grey published and unpublished literature which minimises publication and reporting bias on outcomes [65, 66]; and prior registration of the systematic review with PROSPERO to ensure transparency and rigor, reducing bias in study selection [65].

This systematic review is limited by a number of factors: firstly, concentrating only on clinician interventions notwithstanding the improvement in diagnostic accuracy demands involvement of all stakeholders notably patients and their families; secondly, considering only studies post-1990 and before April 2017 hence results may exclude important earlier and more recent studies; and lastly, methodological limitation since studies only in English language were included (which perhaps could explain some of the bias towards studies from the US).

## Conclusion

In conclusion, we found limited evidence on suggested interventions actually used in clinical settings. There is some evidence that trigger algorithms, including computer based and alert systems, may reduce delayed diagnosis and improve diagnostic accuracy. In trauma settings, strategies such as additional patient review (e.g. trauma teams) reduced missed diagnosis and in radiology departments review strategies such as team meetings and error documentation may reduce diagnostic error rates over time. However, none of the studies explored cost effectiveness in real practice. For this reason, it is recommended that future work establish the effectiveness and cost effectiveness of suggested interventions in real-world clinical settings. The implication is that at both the national and global level, policies around patient safety need to be harmonised to enable comparison and evaluation of progress with time. We agree with Singh and colleagues in highlighting the importance of WHO’s global leadership as instrumental in addressing diagnostic error as a global problem [61]. Policy makers can prioritise patient safety and research to ensure sustainable funding to develop actionable, evidence based interventions to address diagnostic errors, whether due to delayed diagnosis, misdiagnosis or missed diagnosis.

## Additional files

Additional file 1: Search Strategies. (DOCX 17 kb)

Additional file 2: Risk of bias assessment. (DOCX 27 kb)

Additional file 3: The effectiveness of audit and communication strategies in reducing diagnostic errors. (DOCX 21 kb)

## Data Availability

Full electronic search strategies and review protocol are available in Additional file 1.

## Abbreviations

PSNet: Patient Safety Network
RCT: Randomised Controlled Trials
