# Supplementary material for: Strategies to reduce diagnostic errors: a systematic review
Source: BMC Med Inform Decis Mak. 2019 Aug 30;19:174. doi: 10.1186/s12911-019-0901-1 (PMC6716834; doi:10.1186/s12911-019-0901-1)
Supplement: Supplementary file 1 — Search Strategies. (DOCX 17 kb) [file 12911_2019_901_MOESM1_ESM.docx]

**Additional file 1: Search Strategies**

Additional file 1.1: MEDLINE complete and CINAHL complete search strategy

| Step 1 | MW “diagnos* error*” or MW ”mis* diagnos*” or MW “safe* diagnos*” or MW “diagnos* delay*” or MW “delay* diagnos*” |
| --- | --- |
| Step 2 | “Audit* next/2 system*” OR “Audit* next/2 Program*” OR “Audit* near/2 nurs*” OR “Audit* near/2 hospital*” OR “Audit* near/2 emergency department” OR “Audit* near/2 clinic*” OR Intervention* OR “program* near/2 evaluation*” OR “Audit* model*” OR “Audit* record*” OR “Audit* practic*” OR “prevention near/2 diagnostic error*” |
| Step 3 | Communication* near/2 strateg*” OR “system* strateg*” OR “decision support*” |
| Step 4 | 2 or 3 |
| Step 5 | 4 and 1 |

Additional file 1.2: EMBASE search strategy

| Step 1 | MJ 'diagnos* error*' OR TI 'diagnos* error*' OR AB 'diagnos* error*' OR MJ 'mis* diagnos*' OR TI 'diagnos* error*' OR AB 'diagnos* error*' OR MJ 'safe* diagnos*' OR TI 'safe* diagnos* AB 'safe* diagnos*''OR MJ 'diagnos* delay*' OR TI 'diagnos* delay*' OR AB 'diagnos* delay*' OR MJ 'delay* diagnos* OR TI 'delay* diagnos*' OR AB 'delay* diagnos*‘ |
| --- | --- |
| Step 2 | “Audit* next/2 system*” OR “Audit* next/2 Program*” OR “Audit* near/2 nurs*” OR “Audit* near/2 hospital*” OR “Audit* near/2 emergency department” OR “Audit* near/2 clinic*” OR Intervention* OR “program* near/2 evaluation*” OR “Audit* model*” OR “Audit* record*” OR “Audit* practic*” OR “prevention near/2 diagnostic error*” |
| Step 3 | Communication* near/2 strateg*” OR “system* strateg*” OR “decision support*” |
| Step 4 | 2 or 3 |
| Step 5 | 4 and 1 |

Additional file 1.3: PSNet search strategy

| Search phase | “audit systems communication strategies clinical settings clinician misdiagnosis diagnostic error” |
| --- | --- |
| Sort by | Most Recent |
| Page setting | Show 100 per page |
| Resource type | journal articles and Books/Report |
| published work between 2017-1990 | Journal Articles – first 80 pages when sorted by 100 per page  Books/Report – first 5 pages when sorted by 100 per page |

Additional file 1.4: Google Advanced search strategy

| Search phase | “Diagnostic error, communication, audit” |
| --- | --- |
| Published/ unpublished work between 2017-1990 | Hand searched potentially relevant articles |
