# Supplementary material for: Strategies to reduce diagnostic errors: a systematic review
Source: BMC Med Inform Decis Mak. 2019 Aug 30;19:174. doi: 10.1186/s12911-019-0901-1 (PMC6716834; doi:10.1186/s12911-019-0901-1)
Supplement: Supplementary file 2 — Risk of bias assessment. (DOCX 27 kb) [file 12911_2019_901_MOESM2_ESM.docx]

**Additional file 2: Risk of bias assessment**

Additional file 2.1: Risk of bias assessment of RCTs using Cochrane Checklist

| **Lead Author, Year** | **Allocation concealment** | **Blinding - participants and researchers** | **Blinding - outcome assessment** | **Incomplete outcome data** | **Selective reporting** | **Random sequence generation** |
| --- | --- | --- | --- | --- | --- | --- |
| 1. Ely, 2015 ([1](#_ENREF_1)) | High Risk | High Risk | High Risk | High Risk | Low risk | Low risk |
| 1. Meyer, 2016 ([2](#_ENREF_2)) | Low risk | Low risk | Low risk | Low risk | Low risk | Low risk |
| 1. Tsai, 2003 ([3](#_ENREF_3)) | Low risk | Low risk | Low risk | Low risk | Low risk | Low risk |
| 1. Murphy, 2015 ([4](#_ENREF_4)) | Low risk | Low risk | Low risk | Low risk | Low risk | Low risk |
| 1. Schriger, 2001 ([5](#_ENREF_5)) | Low risk | Low risk | Low risk | Low risk | Low risk | Low risk |
| 1. Cannon, 2000 ([6](#_ENREF_6)) | Low risk | High risk | Low risk | Low risk | Low risk | Low risk |
| 1. Sibbald, 2013 ([7](#_ENREF_7)) | Low risk | High risk | Medium risk | Low risk | Low risk | Low risk |
| 1. Boguševičius, 2002([8](#_ENREF_8)) | Low risk | Low risk | Low risk | Low risk | Low risk | Low risk |
| 1. Bergman, 2008 ([9](#_ENREF_9)) | High risk | High risk | Low risk | Low risk | Low risk | Low risk |

Assessed low risk as good quality, medium risk as medium quality and high risk as poor quality.

Additional file 2.2: Risk of bias assessment of non-RCTstudies using Effective Public Health Practice Project quality assessment tool

| Lead Author, Year | Selection bias | Study design | Data collection methods | Withdrawals and drop outs | Final decision |
| --- | --- | --- | --- | --- | --- |
| 1. Singh, 2007 ([10](#_ENREF_10)) | Moderate | Moderate | Strong | Moderate | Moderate |
| 1. Aaland, 1996 ([11](#_ENREF_11)) | Moderate | Weak | Strong | Moderate | Moderate |
| 1. Singh, 2010 ([12](#_ENREF_12)) | Moderate | Moderate | Strong | Moderate | Moderate |
| 1. Perno, 2005 ([13](#_ENREF_13)) | Moderate | Weak | Strong | Moderate | Moderate |
| 1. Selker, 1998 ([14](#_ENREF_14)) | Moderate | Strong | Strong | Moderate | Moderate |
| 1. Graber, 2014 ([15](#_ENREF_15)) | Strong | Moderate | Strong | Strong | Moderate |
| 1. David, 2011 ([16](#_ENREF_16)) | Weak | Weak | Strong | Weak | Weak |
| 1. Ramnarayan, 2006 ([17](#_ENREF_17)) | Weak | Weak | Strong | Moderate | Weak |
| 1. Fridriksson, 2001 ([18](#_ENREF_18)) | Moderate | Moderate | Strong | Weak | Moderate |
| 1. Espinosa, 2000 ([19](#_ENREF_19)) | Moderate | Moderate | Strong | Weak | Moderate |
| 1. Soininen, 2012 ([20](#_ENREF_20)) | Moderate | Weak | Strong | NA | Moderate |
| 1. Howard, 2006 ([21](#_ENREF_21)) | Moderate | Weak | Strong | NA | Moderate |
| 1. Jiang, 2000 ([22](#_ENREF_22)) | Moderate | Weak | Strong | NA | Moderate |
| 1. Medford-Davis, 2015 ([23](#_ENREF_23)) | Moderate | Weak | Strong | Moderate | Moderate |
| 1. Casalino, 2009 ([24](#_ENREF_24)) | Moderate | Weak | Strong | Moderate | Moderate |
| 1. Wellwood, 1992 ([25](#_ENREF_25)) | Moderate | Weak | Strong | Moderate | Moderate |
| 1. Sibbald, 2013 ([26](#_ENREF_26)) | Moderate | Weak | Strong | Moderate | Moderate |

Assessed strong as good quality, moderate as medium quality and weak as poor quality. Note that confounding and blinding have been excluded since they didn’t apply to most of the descriptive studies. NA=Not applicable to study
