# Supplementary material for: Strategies to reduce diagnostic errors: a systematic review
Source: BMC Med Inform Decis Mak. 2019 Aug 30;19:174. doi: 10.1186/s12911-019-0901-1 (PMC6716834; doi:10.1186/s12911-019-0901-1)
Supplement: Supplementary file 3 — The effectiveness of audit and communication strategies in reducing diagnostic errors. (DOCX 21 kb) [file 12911_2019_901_MOESM3_ESM.docx]

**Additional file 3: The effectiveness of audit and communication strategies in reducing diagnostic errors**

| **Communication** | | | |
| --- | --- | --- | --- |
| **Article** | **Intervention/Control diagnostic error** | | **Difference in diagnostic error reduction (Effectiveness)** |
| Cannon, 2000  (RCT) | A higher rate of screening rate for mood disorder 86.5 vs.61% | | 25.5% |
| Singh, 2010 | 18.3% alerts were no-impact alerts  26.4% alerts-no evidence of documented follow-up  6.8% alerts lacked timely follow-up  17.4% alerts were redundant test(unnecessarily ordered) | | - |
| Meyer, 2016  (RCT) | Response to follow-up by email was 11.1% of cases, 72.1% for telephone, and 73.4% for contacting director. | | Delayed follow-up was 88.9% using emails, 31.4% using telephone and 54.5% for contact by clinic directors. |
| Murphy, 2015  (RCT) | Time to diagnostic evaluation (intervention group vs. control group)  Colorectal cancer 104 vs 200 days  Prostate cancer 144 days vs. 192 days Lung 65 vs 93 days  Proportion of patients that receive diagnostic evaluation by primary care physician’s final review 73.4% vs. 52.2% | | Colorectal 96 days  Prostate 48 days  Lung 28 days  Final review diagnostic evaluation 21.2% more patients reviewed |
| Singh, 2007 | Providers failed to acknowledge receipt of over one-third of alerts, 4% of them were abnormal results. | | - |
| Medford-Davis, 2015 | 18.3% of cases were no-impact alerts. 6.8% of total lacked timely follow-up. | | - |
| **Audit** | | | |
| Aaland, 1996 | 13% of delayed diagnosis were found at routine follow-up. | | - |
| Casalino, 2009 | Process score^[[1]](#footnote-1)^  (pg. 1125) | Failure to inform or document (%) | Reduction in error due to the use of all five processes was 13% |
|  | 0-3.0 | 18.1 |  |
|  | 3.1-3.9 | 6.1 |  |
|  | 4.0-5.0 | 5.1 |  |
| Perno, 2005 | Previous incidence of missed injury was 4.3%.  Current incidence 0.46%. | | 3.84% reduction of delayed diagnosis of injuries |
| Espinosa, 2000 | False negative errors declined from 3% to 1.2%, and reduced further to 0.3% after redesigning. | | 2.7% reduction in interpreting errors |
| Howard, 2006 | Tertiary examination before discharge of admitted patient, 14% of patients had missed injuries. | | - |
| Selker, 1998 | Intervention didn’t change appropriate admission to CCU or telemetry units for patients with acute cardiac ischemia (96%)  For patients without the condition, in hospitals with high-capacity CCUs and relatively low-capacity cardiac telemetry units, the intervention reduced CCU admission from 15% to 12%, increased emergency department discharges from 49% to 52%.  Across all hospitals for patients evaluated by unsupervised residents, reduction in CCU admission from 14% to 10%, telemetry unit admissions reduced from 39% to 31%, and increase in discharges to home from 45% to 56%  For patients with stable angina, in hospitals with high capacity CCUs, reduction in CCU admissions from 26% to 13%, increase in home discharges from 20% to 22%.  In hospitals with high capacity telemetry unit, a reduction in admissions from 68% to 59%, and increased discharge from 10% to 21% | | Reduction in admission ranged from 3-8% for patients without ischemia, 9-13% for patients with stable angina  Increase in discharge for patients without ischemia varied from 3-11% and 2-11% for patients with ischemia |
| Tsai, 2003  (RCT) | Without computer interpretation(CI), 48.9% interpreted correctly,  With CI, 55.4% interpreted correctly | | improvement of 6.6% in correct interpretation |
| Bergman, 2008  (RCT) | No significant difference in time or diagnostic variables | | - |
| David, 2011 | Intervention (computer decision support system) had 64% cases of correct diagnosis compared to 14% without intervention | | 50% more correct diagnosis |
| Schriger, 2001  (RCT) | Frequencies of physician psychiatric diagnosis and referral not different between two groups with and without computerised psychiatric interview report results (report 9%, no report 9%, change=0) | | No difference |
| Wellwood, 1992 | The initial accuracy for non-specific abdominal pain (NSAP) increased from 48% with no decision aids provided to 69% with forms, computer and performance feedback. 80% of this improvement due to correct diagnosis of NSAPs instead of no diagnosis being given. | | 21% increase in accuracy for NSAPs |
| Soininen, 2012 | The tool was able to predict conversion of Alzheimer’s disease at an accuracy of 93.6% | | - |
| Boguševičius, 2002  (RCT) | Computer-aided diagnosis had no significant advantage over contrast radiology in accuracy of diagnosis of small bowel obstruction. However, average time period for making the diagnosis was 1 hour for the computer aided group compared to 16 hours for the radiology group. | | No difference in diagnostic accuracy but significant difference in time to diagnosis-15 hours |
| Jiang, 2000 | Average sensitivity for individual radiologists’ review of mammograms without computer aid was 74+-11% and average specificity was 32+-15%.  Average computer-aided sensitivity was 87+-6%, average specificity was 42+-15%  (Sensitivity- the fraction of malignant lesions that received a biopsy recommendation; specificity-the fraction of benign lesions that received a follow-up recommendation) | | Average sensitivity difference 13%; average Specificity difference 10% |
| Fridriksson, 2001 | Initial diagnostic error evident in 12% of patients. Diagnostic errors reduced by 77% as a result of continuous interaction between neurosurgeons and local physicians. | | 77% reduction |
| Graber, 2014 | Checklists prompted consideration of additional diagnostic possibilities, changed the working diagnosis in approximately 10% of cases, and anecdotally was thought to be helpful in avoiding diagnostic errors. | | - |
| Sibbald, 2013 | Experts corrected 19.2% of their errors with a checklist compared with 1.8% without a checklist. | | An increase of 17.4% in correcting errors |
| Ely, 2015  (RCT) | Mean error rate for checklist physicians not significantly different from the rate among usual-care physicians (11.2% vs. 17.8%; p=0.46).  Post-hoc sub group analysis of emergency physicians using the checklist showed a lower mean error rate than emergency physicians in the usual-care group (19.1% vs. 45.0%; p= 0.04) | | No difference in mean error  post-hoc sub group had a reduction in mean error of 25.9% |
| Sibbald, 2013  (RCT) | 46% residents made the correct diagnosis before using the checklist compared with 51% afterwards | | 5% improvement in correct diagnosis |

1. Ranged from a minimum of zero and a maximum of 5. [↑](#footnote-ref-1)
